# Impact of Alterations in Homocysteine, Asymmetric Dimethylarginine and Vitamins-Related Pathways in Some Neurodegenerative Diseases: A Narrative Review

**DOI:** 10.3390/ijms26083672

**Published:** 2025-04-13

**Authors:** Caterina Saija, Monica Currò, Riccardo Ientile, Daniela Caccamo, Maria Paola Bertuccio

**Affiliations:** Department of Biomedical and Dental Sciences and Morpho-Functional Imaging, University of Messina, 98125 Messina, Italy; caterina.saija@unime.it (C.S.); monica.curro@unime.it (M.C.); riccardo.ientile@unime.it (R.I.); daniela.caccamo@unime.it (D.C.)

**Keywords:** hyperhomocysteinemia, asymmetric dimethylarginine, vitamins, Parkinson’s disease, Alzheimer’s diseases, multiple sclerosis

## Abstract

Hyperhomocysteinemia (HHcy) influences the development and progression of neurodegenerative disorders in different ways. Homocysteine (Hcy) metabolism is related to that of asymmetric dimethylarginine (ADMA) and group B vitamins. The breakdown of the pathway involving nitric oxide (NO) and ADMA can be considered one of the causes of endothelial alteration that represents a crucial step in the development of several neurodegenerative disorders. Deficiencies of vitamins other than group B ones, such as D and A, have also been associated with central nervous system disorders. The aim of this narrative review is to describe the link between HHcy, ADMA, and vitamins in Parkinson’s disease (PD), Alzheimer’s disease (AD), and multiple sclerosis (MS) in terms of dysfunctional pathways and neuropathological processes, performing a literature search from 2015 to 2025 on PubMed. This review also provides an overview of the effects of vitamin supplementation on neurodegenerative diseases. The alteration of pathways involving NO production can lead to HHcy and elevated ADMA concentrations, causing neurodegeneration through various mechanisms, while vitamin supplementation has been shown to reduce Hcy levels, although with conflicting results about the improvement in clinical symptoms. Further studies are needed to develop optimal combined therapeutic strategies.

## 1. Introduction

Endothelial dysfunction represents a crucial event in the development of several neurodegenerative diseases. The breakdown of the pathway involving nitric oxide (NO), NO synthase (NOS), and asymmetric dimethylarginine (ADMA) can be considered one of the causes of endothelial alteration [1].

**Figure 1:**
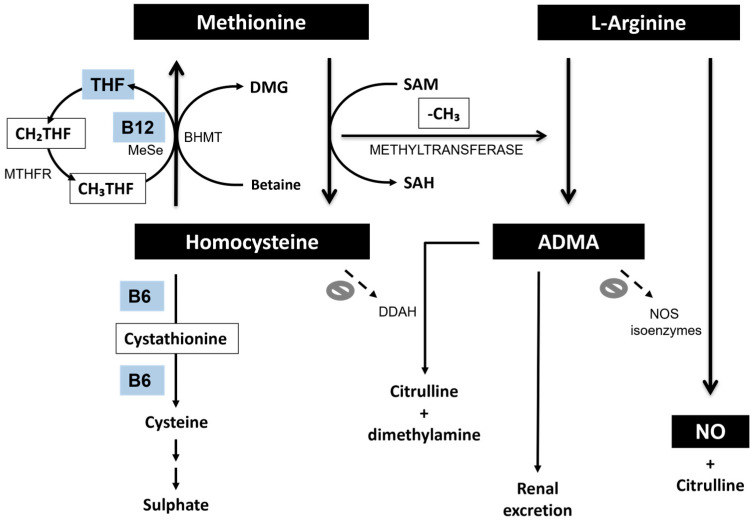
Relationships between methionine–homocysteine metabolism and ADMA and NO synthesis pathways. THF, tetrahydrofolate; CH₂THF, 5,10-methylenetetrahydrofolate; CH₃THF, 5-methyltetrahydrofolate; MTHFR, methylenetetrahydrofolate reductase; MeSe, methionine synthase; BHMT, betaine-homocysteine methyltransferase; DMG, dimethylglycine; SAM, S-adenosyl methionine; SAH, S-adenosyl homocysteine; ADMA, asymmetric dimethylarginine; DDAH, dimethylarginine dimethylaminohydodrolase; NOS, nitric oxide synthase; NO, nitric oxide.

Worldwide, extensive clinical research has been carried out to establish the possible causative role of supraphysiological total plasma homocysteine (tHcy) levels in the development of vascular and neurodegenerative diseases, including coronary heart disease, stroke, and brain atrophy [2]. It is already known that homocysteine (Hcy) metabolism is associated with that of ADMA productions [3], coming from the breakdown of proteins containing methylated arginine residues [4,5], which in turn are formed after the post-translational transfer of methyl groups from S-adenosyl methionine (SAM) [6]. Based on evidence about methylation/demethylation, SAM is considered to be the main donor of methyl groups in numerous metabolic processes, leading to the formation of methyl derivatives. Methyl transfer reactions generate S-adenosylhomocysteine (SAH), and its subsequent hydrolyzation produces adenosine and Hcy. The cellular “methylation capacity” or SAM-to-SAH ratio depends on the intracellular concentrations of both SAM and SAH [7]. A dysregulation of methyltransferase reactions leads to the accumulation of Hcy [8]. In addition, two equivalents of Hcy are formed as a by-product of ADMA synthesis, which requires the transfer of two methyl groups [9]. In turn, Hcy inhibits the dimethylarginine dimethylaminohydrolase (DDAH)-mediated degradation of ADMA, causing ADMA accumulation and NO synthesis inhibition [10]. This explains, at least in part, how hyperhomocysteinemia (HHcy) is involved in the impairment of the endothelial vasodilator function (Figure 1).

The observation that ADMA is one of the products methylated by SAM may also be confirmed by the evaluation of other pathways associated with SAM metabolism: transmethylation, transulfuration, and transaminopropylation [11]. Transmethylation plays several roles in cell biology since it is involved in brain development, even if this process is based on a balance between neurogenesis and neurodegeneration that lasts a lifetime. Several studies have reported a link between the development of severe neuropsychiatric diseases throughout the life and the breakdown of transmethylation pathways with deficient SAM production [11,12].

**Figure 2:**
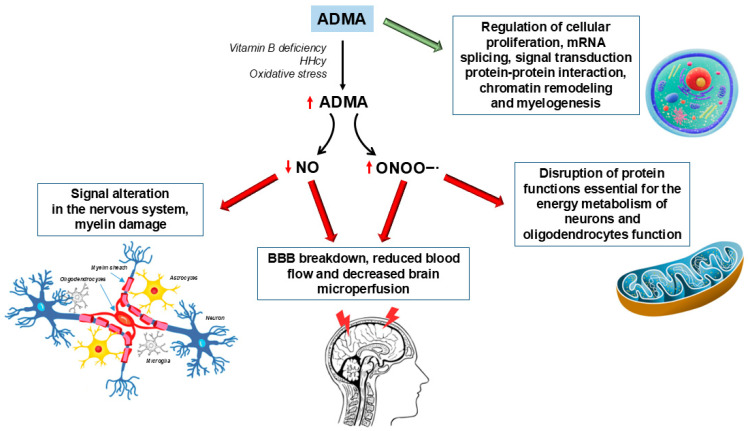
Effects of physiological and supraphysiological concentrations of ADMA on biological systems. ADMA, asymmetric dimethylarginine; BBB, blood–brain barrier; HHcy, hyperhomocysteinemia; NO, nitric oxide; ONOO−∙, peroxynitrite.

Vitamins B9, B12, and B6 are necessary for the methylation of substrates useful to generate ADMA. Vitamin B12 and B9 are involved in the methionine–homocysteine cycle; thus, low levels of these vitamins may cause HHcy, leading to endothelial dysfunction and vascular disease [1,13] (Figure 2).

A dysregulation of the pathways mentioned above can result in the elevation of serum levels of Hcy and ADMA and a reduction in folic acid and methionine concentrations, leading, in turn, to the development of a general pro-atherosclerotic state caused by Hcy-induced impairment of redox balance and ADMA-mediated inhibition of NO synthesis [14] (Figure 2).

The aim of this narrative review is to report the link between HHcy, ADMA, and vitamins in Parkinson’s disease (PD), Alzheimer’s disease (AD), and multiple sclerosis (MS) in terms of dysfunctional pathways and neuropathological processes. Additionally, an overview of the effects of vitamin supplementation in the above-cited neurodegenerative diseases will be provided.

## 2. Methods

For this narrative review, we performed a literature search from 2015 to 2025 on PubMed using the following search terms (individually and/or in combination):

“Homocysteine and hypovitaminosis in Parkinson”, “Asymmetric dimethylarginine in Parkinson”, “Homocysteine AND hypovitaminosis in Alzheimer”, “asymmetric dimethylarginine and hypovitaminosis in Alzheimer”, “Homocysteine and hypovitaminosis in Multiple Sclerosis”, and “Asymmetric dimethylarginine in Multiple sclerosis”.

The inclusion criteria were (1) study type: reviews and experimental studies, (2) language: English, and (3) topic: relevance in the field of this narrative review.

**Figure 3:**
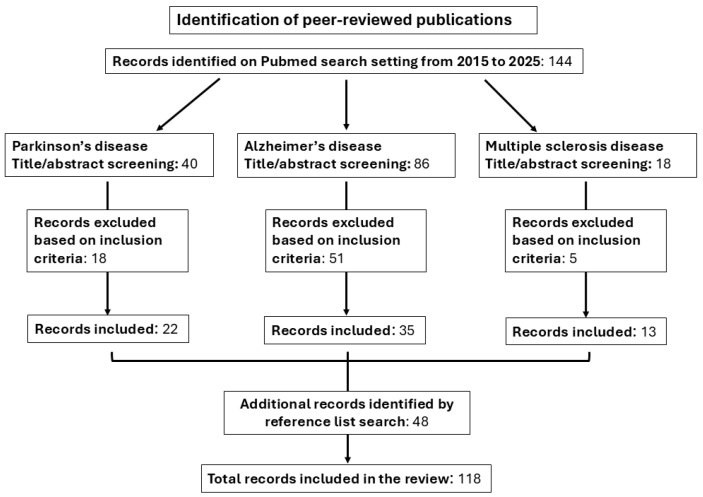
Flow chart describing the process used to identify and select relevant publications.

As shown in Figure 3, our search yielded a total of 144 publications: 40 about Parkinson’s disease, 86 about Alzheimer’s disease, and 18 about multiple sclerosis. The titles and abstracts of all the manuscripts were screened by 2 reviewers and selected based on the inclusion criteria. Duplicates were also eliminated.

The reference lists of the selected articles were also checked to find further relevant research. Opinion and editorial references were not considered.

## 3. Homocysteine and Hypovitaminosis in Parkinson’s Disease

PD is a geriatric neurodegenerative disorder characterized by α-synuclein deposits, resulting in a degenerative movement disorder [15,16,17].

It is already well known that high Hcy levels represent a risk factor for PD [18,19], but how Hcy is involved in the molecular mechanisms causing neurotoxicity has yet to be defined.

Tomic and coworkers [17] reported that elevated Hcy levels in PD patients could be a consequence of levodopa therapy or a low vitamin B12 level. With regard to the first hypothesis, Hcy derives from the hydrolysis of SAH resulting from levodopa catabolism by the enzyme catechol-O-methyltransferase (COMT) [20,21]. Otherwise, the authors found out that Hcy level strongly negatively correlates with vitamin B12 (rs = −0.519; *p* < 0.002) and folic acid (vitamin B9) (rs = −0.502; *p* < 0.003). Similar results were obtained by Liang Shen meta-analyses [16], showing that the vitamin B12 level was lower in PD patients than in matched controls, and that a high dietary intake of vitamin B6 may be associated with a decreased risk of PD, while there is no evidence to support the same effects of vitamin B12 and folate. On the contrary, a recent clinical study reported that vitamin B12 and folate supplementation may decrease Hcy levels in PD patients and, in parallel, their deficiency is associated with the accumulation of Hcy [22].

Folate supplementation and the consequent decrease in the Hcy level were also associated with the prevention of ischemic stroke in PD patients treated with orally administered levodopa [23]. Elderly PD individuals with gastrointestinal dysfunction showed a decrement in folate biosynthesis and riboflavin metabolism and an increment in the microbial production of Hcy in the gut. Folic acid deficiency could lead to HHcy in PD probably because of the failure of its remethylation and conversion to methionine in a reaction dependent on folate metabolism and vitamin B12 [24].

A recent study characterized the human metabolome of subclinical vitamin B12 deficiency in elderly adults [25]. In this trial, the treatment with vitamin B12, consisting of one injection of 10 mg vitamin B12, was associated with an increment in the metabolic markers involved in myelin integrity and membrane dynamics, such as plasmalogens that are ether phospholipids involved in PD and other neurological disorders [26,27], suggesting novel mechanisms connecting vitamin B12 to neuroprotection. Furthermore, the authors recorded a significant decrement in tHcy and methylmalonic acid (MMA) after the treatment, while no change was observed in serum folate. Correlation analysis showed that serum vitamin B12 was directly correlated with targeted metabolomic variables, while tHcy and MMA were inversely correlated.

The kynurenine (Kyn) pathway is associated with oxidative stress and neuroinflammation that contribute to PD pathophysiology [28]. Moreover, it has been widely demonstrated that the Kyn pathway is dysregulated in PD. Metabolomic studies have detected lower levels of kynurenic acid and a reduced kynurenic acid/kynurenine ratio, higher levels of quinolinic acid, and an altered quinolinic acid/kynurenic acid ratio in the plasma and cerebrospinal fluid of patients with PD [29,30,31].

A clinical case study of a woman with PD [32] underlined that severe vitamin B6 deficiency can trigger HHcy and induce extreme alterations in the Kyn pathway. In fact, the active form of vitamin B6, pyridoxal 5-phosphate (PLP), is a cofactor of the cystathionine beta synthase enzyme, and it is also necessary to convert kynurenine in kynurenic acid (KA) by the kynurenine aminotransferase (KAT) enzyme and in anthranilic acid (AA) by kynureninase (KYNU). Furthermore, PLP is involved in the conversion of 3-hydroxykynurenine (HK) to xanthurenic acid (XA) by KAT or to 3-hydroxyanthranilic acid (HAA) by KYNU.

The blood Hcy level is also influenced by riboflavin (vitamin B2), since 5,10-methylenetetrahydrofolate reductase (MTHFR), the key enzyme in the folate/homocysteine pathway, is a flavin adenine dinucleotide (FAD)-dependent enzyme [15]. At base position 677 of the *MTHFR* gene, a genetic polymorphism in which cytosine is replaced by thymine results in dramatically reduced enzymatic activity [33]. As already reported [34], a high riboflavin intake can prevent the negative effects of the genetic variant *MTHFR* C677T and decrease Hcy levels. Furthermore, the T mutated allele has also been reviewed as a predisposing factor for the development of PD in European subjects [35].

## 4. ADMA in Parkinson’s Disease

ADMA is a small water-soluble compound that, at uremic concentrations, can be associated with many diseases. This nonproteinogenic amino acid is an endogenous competitive inhibitor of NOS that is involved in the synthesis of NO, acting as a signaling molecule in the nervous system. For this reason, ADMA may be associated with PD pathogenesis and progression. Elevated concentrations of ADMA have been found in the cerebrospinal fluid (CSF) of PD patients and can be associated with a reduction in blood flow and a decrease in brain microperfusion [36] (Figure 2). In parallel, the plasma levels of both ADMA and NO in PD patients were significantly higher than those in healthy controls, and these increments were significantly correlated with plasma Hcy levels [1]. It has been hypothesized that supplementation with B vitamins (B9, B6, and B12) could be an efficient way to reduce Hcy levels, and consequently ADMA levels, by reducing the generation of ADMA from protein breakdown and/or reducing the homocysteine-mediated inhibition of DDAH activity. However, contradictory results have been reported [9,37].

## 5. Homocysteine and Hypovitaminosis in Alzheimer’s Disease

Plasma HHcy has been reported as an independent risk factor for cognitive decline and dementia [38,39,40]. Cognitive impairment due to an elevated plasma Hcy concentration has been associated with silent cerebral infarcts and atherosclerosis [41,42]. In fact, neuronal cell damage and hippocampal atrophy were shown to be resulting from the Hcy-induced activation of N-methyl-D-aspartate receptors [42]. HHcy also causes an increase in amyloid β (Aβ) deposits and the impairment of neurotransmitter synthesis, lowering the potential for hypomethylation [38].

One of the earliest symptoms of cerebrovascular dysfunction associated with AD is endothelial dysfunction [43]. To maintain hemodynamic responses, endothelial cells release endogenous mediators, i.e., endothelin (ET) and NO. In recent years, it has been suggested that cerebrovascular dysfunction in AD is an indirect consequence of Aβ deposition and the increased production of ET1, causing a chronic reduction in cerebral blood flow [43,44]. Endothelial dysfunction could also be caused by the increased production of Hcy oxidation products that leads to the excessive sulfation of connective tissue [45].

In addition, HHcy has been associated with an alteration of several crucial methylation reactions occurring in the brain, including DNA methylation, catecholamine and phospholipid synthesis, and the hyperactivation of kinases with an increment in tau phosphorylation [46].

High Hcy and low or even subclinical B vitamin concentrations have been associated with cognitive decline [47,48,49]. Methionine synthase is a vitamin B12 and folate-dependent enzyme that remethylates Hcy to methionine. It is therefore not surprising that B vitamins have the ability to lower plasma Hcy concentrations, improving cognitive function in elderly individuals with or without dementia, and play an important role in the prevention of AD. However, it must be highlighted that vitamin intervention trials have not been conclusive; in fact, several, but not all, vitamin trials and observational studies have documented an improvement in cognitive functions [38,50,51]. Indeed, dietary intervention with vitamins B12 and folate supplementation seems to decrease blood Hcy levels by improving clinical outcomes in Mild Cognitive Impairment (MCI), but similar results were not obtained in AD populations [52,53].

Together with vitamins B, also vitamin D and the cholesterol-oxidized metabolite 27-hydroxycholesterol (27-OHC) play important roles in AD. Vitamin D deficiency influences CYP27A1-related gene and protein expressions that may be involved in the mechanism of learning and memory impairment. Recent findings suggest that a co-supplementation of vitamin B12 and folic acid with vitamin D is able to significantly reverse this effect by influencing CYP27A1 expression and regulating the metabolism of 25-hydroxyvitamin D (25(OH)D), 27-OHC, and SAM [54].

Epigenetic modifications mediate AD onset and progression; in particular, the Presenilin 1 (*PS1*) gene is a specific locus of demethylation in AD patients. Using an *APP/PS1* (Amyloid Precursor Protein/Presenilin 1) transgenic mouse model of AD (mice with APPswe/PS1ΔE9 Mutations), Wen Li and coworkers [55] found that supplementation with folic acid and/or SAM increases methylation potential and DNMT activity, modifies DNA methylation, and decreases APP, PS1, and Aβ protein levels. Similarly, Shoudan Sun and colleagues [56] showed that folic acid and SAM significantly repressed Hcy-induced neurodegenerative injury by modulating PS1 and protein phosphatase 2A (PP2A) methylation levels. PP2A catalyzes the tau dephosphorylation reaction, and the downregulation of its promoter methylation in the brain of AD subjects can accelerate tau phosphorylation [57].

In the brain of AD patients, there is an accumulation of extracellular plaques characterized by Aβ as well as intracellular neurofibrillary tangles composed of hyperphosphorylated tau. These pathological changes can also lead to visual dysfunction. Plasma HHcy induces AD-like Aβ accumulation and tau hyperphosphorylation in rats [20,21,58,59]; thus, a reduction in plasma Hcy concentration could be used as strategy to arrest the AD process. Supplementation with folate and vitamin B12 efficiently reduces plasma Hcy levels with the attenuation of AD-like pathologies in the retina [60]. Jing Guo and colleagues [61] suggested that elevated Ser262 hyperphosphorylated tau (pS262-tau), the decreased methylation of methylated protein phosphatase-2A catalytic subunit (M-PP2Ac), and increased de-methylated PP2Ac (DM-PP2Ac) in retina can be used in the early stage of HHcy-induced AD as surveillance biomarkers for diagnosis.

Together with Aβ deposits, increased fibrinogen levels were found in the blood vessels and brain parenchyma of AD patients with HHcy. It seems that Hcy and its metabolite, Hcy thiolactone (HCTL), promote the interaction between fibrinogen and Aβ, leading to the formation of tighter fibrin clots, thus delaying clot fibrinolysis [62].

HHcy amplifies Aβ 40-dependent activation of the death receptor 4 and 5 (DR4/5)-mediated extrinsic apoptotic pathway in human cerebral endothelial cells (cECs) by modulating DR4/5 expression, caspase 8/9/3 activation, DNA fragmentation, and cytochrome c release. In particular, Aβ40-E22Q (AβQ22; vasculotropic Dutch mutant) promotes DR4/5-mediated apoptosis in cECs and consequently increases blood–brain barrier (BBB) permeability and angiogenic impairment [63].

The fact that HHcy induces gene expression changes is also proved by its ability to influence the expression levels of 5-lipoxygenase (5LO), the promoter of which is regulated by methylation, resulting in gene upregulation in AD patients. Recent findings have shown that DNA hypomethylation is linked to elevated SAH levels and Hcy-dependent 5LO activation and Aβ formation [64,65].

Comparing metabolic profiles between HHcy patients and healthy control groups, Xinshu Zhao and colleagues [66] identified alterations in amino acid metabolism (specifically arginine, alanine, glutamate, and aspartate), pyruvate metabolism, and the tricarboxylic acid (TCA) cycle in subjects with HHcy, suggesting that dysregulation of these metabolic pathways can influence the onset and progression of AD.

A more in-depth analysis of the biochemical processes involved in HHcy-induced endothelial injury revealed that HHcy increases glucose uptake and oxidation through the stimulation of reactive oxygen species (ROS) production in mitochondria, glycogen degradation, and NAD+/CoA synthesis [67]. The HHcy-mediated activation of inflammatory responses occurs through the induction of inflammasome pyroptosis, IL-1β, and adhesion molecules, as well as adenylate cyclase-modulating G-protein-coupled receptor pathways and interferon α/β signaling. Furthermore, HHcy is involved in the activation of the ubiquitin proteasome system and lysosome autophagy, leading to cell degradation. Finally, HHcy blocks the cell cycle at G1/S and S/G2 transitions, inhibits proliferation through miRNA335/Vasohibin 1 and other axes, and arrests the spindle checkpoint complex and cytokinetic abscission [67].

Finally, it is noteworthy that the Apolipoprotein E (ApoE) genotype may be associated with the development of HHcy and that the Ɛ4 allele represents a risk factor for vascular contributions to cognitive impairment and dementia (VCID), and it is also considered to be the strongest genetic risk factor for late-onset AD [68]. ApoE is a protein that binds and transports cholesterol into the brain and peripheral tissues; ApoE Ɛ4 carriers showed alterations in the BBB with increased permeability and elevated levels of matrix-metalloproteinases, probably due to the related HHcy [69].

## 6. ADMA in Alzheimer’s Disease

The plasma ADMA level when increased in AD patients may contribute to its pathogenesis by modulating the critical functions of the BBB (Figure 2). Moreover, an inverse correlation was found between the concentration of NO in the CSF and the degree of cognitive impairment, thus underlining a possible neuroprotective effect of NO in AD [70,71,72].

In AD patients, the production of the enzyme NOS is uncoupled, causing a decrease in NO-mediated vasodilation, and the onset of oxidative stress through the production of peroxynitrite species (ONOO−∙) [73]. ADMA causes enzymatic dysfunction binding NOS as a noncanonic ligand [74].

Clemons and colleagues [73] showed that the novel protein arginine methyltransferase 4 (PRMT4) enzyme and ADMA (its downstream product) played a pathological role influencing cerebral blood flow (CBF) in AD. In fact, they proved that the inhibition of PRMT4 in mice bearing tau- and Aβ -producing transgenic mutations (3xTg-AD) resulted in a restored CBF, an increment in NO metabolite production, and also an increase in the functional measurements of NOS uncoupling (reduced 3-nitrotyrosine).

A metabolomic analysis of NO/L–arginine pathway metabolites in dementia showed a decrement in arginine, Arg/ADMA, ADMA, and citrulline levels, while symmetric dimethylarginine (SDMA) and dimethylamine (DMA) resulted in being increased, but this elevation is associated with old age rather than with dementia. These alterations are linked to structural brain changes and worse cognitive impairment [75].

## 7. Homocysteine and Hypovitaminosis in Multiple Sclerosis

MS is an autoimmune condition characterized by chronic inflammation of the CNS. The pathological features of MS are demyelination presenting with plaques or lesions in the CNS, loss of axons, inflammation, and BBB damage [76].

To date, the etiology of MS remains unclear due to its multifactorial nature. However, elevated concentrations of antioxidants and vitamins (vitamin D, E, A and B group) seem to reduce oxidative stress and the inflammatory response in the autoimmune process, decreasing the risk of MS [77]. Riccio and Rossano [78] demonstrated that the prevalence of anti-inflammatory foods in the diet, along with the exogenous integration of some food supplements, could be useful to reduce the release of pro-inflammatory compounds and maximize the effects of immunomodulatory drugs in order to slow down disease progression.

Vitamin B12 plays fundamental roles in central nervous system (CNS) function; in fact, it contributes to the formation of the myelin sheath, exerts an immunomodulatory effect, and is an important cofactor for the activity of methionine synthase [77,79,80]. The literature shows conflicting results about vitamin B12 and Hcy levels since some authors reported that deficiencies in vitamin B12 and folate were accompanied by the elevation of Hcy levels, while others did not observe this association [81,82,83,84]. For this reason, more research is necessary to determine whether treatment with vitamin B12 supplements delays disease progression.

HHcy is considered a marker of cell damage, oxidative stress, and neurodegeneration, as well as of folate metabolism functional disorders [81,85,86].

Several experimental studies published in the last decade reported elevated plasma Hcy levels in MS patients compared to healthy individuals [87,88,89]. In particular, a case–control study pointed out higher tHcy levels and a greater frequency of HHcy in Chinese MS patients compared to the control groups, accompanied by lower vitamin B12 levels. Instead, no significant differences were observed in folate levels. The authors suggested that both Hcy and vitamin B12 may play a role in the pathogenesis of MS, with vitamin B12 potentially correlating with disease relapses [89].

Moreover, the high predictive value of elevated tHcy levels in children with early-onset MS compared to healthy peers of the same age has been demonstrated [87]. However, the increase in tHcy in this study cohort was not associated with deficiencies in vitamins B6, B9, or B12, suggesting the potential involvement of polymorphic variants in folate cycle genes.

To further analyze MS progression, Jamroz-Wiśniewska and colleagues recruited 118 MS patients with either a secondary progressive (SP) or relapsing–remitting (RR) clinical course [90]. Notably, tHcy levels were significantly higher in RR-MS and SP-MS patients compared to healthy controls. Treatment with cladribine, a drug known for its potent neuroprotective effects, significantly reduced tHcy levels and increased total serum antioxidant activity only in SP-MS patients [90].

According to this, HHcy was associated with higher disability progression in MS patients, which was evaluated in parallel through the use of specific questionnaires, such as the Multiple Sclerosis Severity Score (MSSS) and Expanded Disability Status Score (EDSS) [88,90]. These findings emphasize the importance of reducing tHcy levels and enhancing serum antioxidant capacity in helping to prevent MS progression.

Vitamin D levels can influence MS clinical management due to the vitamin’s anti-inflammatory and immunomodulatory properties on the activity of B, Th1, and Th17 lymphocytes [91,92,93,94]. There is sufficient evidence that low concentrations of vitamin D are associated with a high risk of developing MS or the relapse of symptoms [95,96,97,98]. Moreover, the level of 25(OH)D negatively correlated with the disability and severity scores of the disease (EDSS and MSSS), as well as Herbert’s six severity grades [99,100]. To date, several epidemiological studies have reported conflicting findings regarding the correlation between the frequency of gene polymorphisms in enzymes of the vitamin D pathway (VDR, CYP27B1, CYP27A1, CYP24A1, CYP2R1, RXR- α, Klotho) and the risk of developing MS [101,102]. To better clarify the risk factors associated with MS, comprehensive genome-wide studies should incorporate environmental influences along with other key factors, such as cofactors, enzyme activity, and the epigenetic regulation of genes involved in vitamin D metabolism [103,104].

Vitamin A supplement seems to be useful in the prevention and treatment of MS. Retinoic acid (RA), a reactive form of vitamin A, is fundamental for neuronal plasticity and regeneration in CNS. Moreover, in order to increase immunotolerance, RA plays a key role in re-establishing the balance between pro-inflammatory (Th1, Th17) and immuno-protective cells (Th2, Treg), modulating B cell and dendritic cell activity [105]. In this regard, studies have pointed out how vitamin A could improve cognitive ability and counteract disability in the trunk upper extremity of MS patients, along with gene expression modulation [106].

## 8. ADMA in Multiple Sclerosis

ADMA and other derivatives play a crucial role in cellular proliferation, mRNA splicing, signal transduction, protein–protein interaction, chromatin remodeling, and myelogenesis (Figure 2). In this regard, an in vivo study revealed that mono- and dimethyl-lysine and ADMA residues on myelin basic protein (MBP) were elevated in the experimental model of MS. These modifications could serve as potential markers of myelin damage and severity of MS neurological disability [107].

Moreover, arginine-methylated proteins can directly or indirectly reduce NO production [108], and several findings associated NO over-production and nitrosative stress with the development of MS [109]. In fact, Th1 and Th17 cells, involved in the pro-inflammatory response in MS, release cytokines that stimulate inducible NOS (iNOS) activity in the CNS [110]. The elevated NO levels contribute to the formation of free radicals and reactive nitrogen species, which can disrupt the function of proteins essential for the energy metabolism of neurons and oligodendrocytes [111].

Six out of the eight studies conducted in the last decade highlighted the elevation of ADMA levels in the serum of MS patients compared to the control group [107,112,113,114,115,116]. On the contrary, the study of Bystrická and coworkers reported significantly lower variations in total methionine and glutathione levels in MS patients compared to the controls, while no differences were found between the two groups in terms of ADMA, Hcy, and total cysteine concentrations, even when dividing patients into the two disease subtypes, RR-MS and SP-MS [117]. No significant differences were found in a clinical trial comparing the serum level of ADMA between RR-MS and healthy groups. However, the use of alpha-lipoic acid (ALA), a potent antioxidant molecule, as an experimental treatment in patients, was able to significantly decrease (*p* = 0.04) the plasma levels of ADMA, pointing out the role of inflammation and oxidative stress in MS development [118].

According to this, particularly noteworthy are the findings from an in vivo study on mice with experimental autoimmune encephalomyelitis (EAE), an animal model of MS, induced by auto-immunization with myelin oligodendrocyte glycoprotein (MOG) and BBB disruption via pertussis toxin [116]. Daily ADMA administration aggravated BBB disruption in EAE mice, worsening both clinical symptoms and CNS disease. Moreover, ADMA treatment alone was sufficient to induce BBB breakdown and EAE disease in MOG-immunized mice, even without pertussis toxin treatment. These findings suggest a critical role for ADMA in BBB dysfunction in EAE and highlight ADMA-mediated mechanisms as potential therapeutic targets for MS [116].

Notably, the research of Monti and coworkers assessed the effects of ADMA and ET1 on cerebral circulation time. They found out that both plasma ADMA and ET1 levels were significantly higher in MS patients than in the control group, without a statistically significant difference between RR-MS and SP-MS subgroups for ADMA concentrations (*p* = 0.6671) [114]. Moreover, the cerebral circulation time was about two times lower in MS patients than in the controls (*p* < 0.0001) and significantly slower in SP-MS than in RR-MS (*p* = 0.0215). The authors supposed that high ET1 and ADMA levels derive from a protective response to early insults aimed at counteracting excessive NO production, whereas persistent pathological ET1 and ADMA levels result in detrimental long-term effects due to increased brain microvessel resistance.

In contrast to this study, SP-MS patients had higher levels of ADMA compared to RR-MS in an MS population recruited by Onmaz and colleagues [115]. In particular, by using the citrulline/Arg ratio as an index of NO levels and NOS activity, they found that the citrulline/Arg ratio was significantly higher in the MS group compared to the controls, according to the current literature [109,110], along with serum ADMA levels. Since ADMA is metabolized to citrulline and DMA by the enzyme DDAH, increased ADMA levels in MS patients could result from reduced DDAH activity and increased PRMT-1 activity in response to oxidative and nitrosative stress. This metabolic rearrangement could explain relatively higher levels of ADMA in circulation, not accompanied by lower concentrations of NO levels. Thus, ADMA, which is an endogenous inhibitor of constitutive and inducible NOS isoenzymes, is not necessarily involved in MS as an inhibitor of NO synthesis [115].

Noteworthy is the research of Haghikia and coworkers [113], in which the authors compared the ADMA concentrations in the serum and CFS of patients affected by MS, neuromyelitis optica (NMO), and other neurologic diseases (ONDs) with those of healthy controls (HCs). They found that the ADMA levels were higher in MS (551 ± 23 nM, *p* = 0.004) and NMO (608 ± 51 nM, *p* = 0.006) patients than in HCs (430 ± 24 nM). Notably, this finding was confirmed in CSF samples, comparing MS (685 ± 100 nM in RRMS; 597 ± 51 nM in SPMS) with OND (514 ± 37 nM; *p* = 0.003) patients, but also comparing SPMS patients and a control group (*p* < 0.05) [112].

However, no significant differences between MS patients and HCs were found in the serum concentrations of nitrate (38.1 ± 2.15 vs. 38.1 ± 3.02 µM, *p* = 0.763) and nitrite (1.37 ± 0.09 vs. 1.55 ± 0.03 µM, *p* = 0.476), as well as between MS and OND patients in CSF concentrations of nitrate (11.3 ± 0.56 vs. 10.5 ± 0.32 µM, *p* = 0.201) and nitrite (2.84 ± 0.32 vs. 2.41 ± 0.11 µM, *p* = 0.767) [113].

## 9. Strengths and Limitations

The main strength of this review is its novelty since it is the first paper that reports the link between Hcy, ADMA, and vitamins in neurodegenerative diseases, providing an accurate overview of the current scientific knowledge on this topic (Table 1).

Moreover, our narrative review highlights the need for additional studies to evaluate how alterations in Hcy and ADMA metabolism influence the onset and progression of neurodegenerative diseases, and whether vitamin supplementation effectively improves disease management.

Regarding limitations, one is that we set a ten-year time limit for the search of articles, even though this selection allowed us to achieve an up-to-date review. Another limitation is represented by having restricted the overview of published works only to the three major neurodegenerative disorders, without also paying attention to minor ones, such as amyotrophic lateral sclerosis, Huntington’s disease, and spinal cord injury, to cite a few. Finally, we used PUBMED as the only database for our research.

## 10. Conclusions

As previously mentioned, extensive research supports the idea that elevated levels of Hcy and ADMA serve as risk factors for cerebrovascular and neurodegenerative diseases. HHcy may worsen neurological dysfunction, directly inducing neuron degeneration and indirectly modulating signaling pathways related to ADMA metabolism. Numerous epidemiological studies and clinical investigations have linked high HHcy and elevated ADMA concentrations to pathological conditions, such as PD, AD, and MS. Experimental research further supports this hypothesis, showing that these metabolites may cause cellular damage through a variety of mechanisms, including the generation of ROS and reactive nitrogen species and inflammatory response.

In neurodegenerative diseases, it is crucial to focus on dietary factors that provide adequate amounts of vitamin B12, B6, and folic acid, which help to alleviate oxidative stress and inflammatory processes. The supplementation of vitamin B6, B12, folic acid, and antioxidants can restore the physiological Hcy metabolism and be helpful in the prevention and treatment of the above-cited diseases.

A significant correlation between serum Hcy and ADMA levels has been reported in PD patients. Accordingly, it may be hypothesized that supplementation with B vitamins might effectively decrease Hcy levels, mitigating its toxic effects, including protein breakdown and the inhibition of DDAH activity, leading to a reduced production of ADMA.

However, despite a substantial body of evidence, conflicting results have been reported regarding the effectiveness of vitamin supplementation in improving these neurodegenerative diseases, even though it has been shown to reduce Hcy levels. Interestingly, not only folate and vitamin B12, which are directly involved in Hcy metabolism, but also other vitamins, such as vitamin D and vitamin A, may play a role in preventing or slowing down the progression of certain neurodegenerative diseases.

Therefore, considering the link between Hcy and ADMA, and their involvement in the onset and progression of neurodegenerative disorders, further studies are necessary to develop optimal combined therapeutic strategies, taking into account the potential benefits of vitamin supplementation in treating these diseases.

## Figures and Tables

**Table 1:** Connection of vitamins with Hcy and ADMA pathways and related impact on Parkinson’s disease, Alzheimer’s disease, and multiple sclerosis.

Vitamins	Parkinson’s Disease	Alzheimer’s Disease	Multiple Sclerosis
Vitamin B12	Vitamin B12 was deficient in PD patients [16,25] and correlated negatively with tHcy levels [1,17,26,27]. Supplementation could have positive effects in reducing tHcy, ADMA levels, and in neuroprotection [22,26,27] or be ineffective [9,16,37].	Reduced B12 concentrations were associated with cognitive decline [47,48,49]. Supplementation in AD patients could be beneficial in reducing tHcy, improving cognitive functions and visual impairments [38,50,51,60], or be ineffective [52,53].	Vitamin B12 levels in MS patients were associated with HHcy and disease relapses [89], but other studies did not observe this association [81,82,83,84,87].
Vitamin B9	HHcy correlated negatively with B9 levels [1,17]. Supplementation could have positive effects on reducing tHcy, ADMA levels, and vascular events [22,23] or be ineffective [9,16,37].	Reduced B9 concentrations were associated with cognitive decline [50]. Supplementation in AD patients could be beneficial in reducing tHcy, APP, PS1, and Aβ protein levels, improving neuronal and visual functions [38,50,51,55,56,60], or be ineffective [52,53].	No significant differences were observed compared to the control group and in children with early-onset MS [87,89].
Vitamin B6	It was deficient in PD subjects [16,32]. Dietary intake of vitamin B6 exhibited preventive effect of developing PD [16] or be ineffective [9,37].		No significant differences were observed in children with early-onset MS [87].
Vitamin B2	Supplementation could neutralize the effects of the MTHFR C677T polymorphism and decrease Hcy levels [34,35].		
Vitamin D		Vitamin D deficiency may be involved in learning and memory. Co-supplementation with vitamins B could have positive effects on vitamin D and SAM metabolism [54].	Evidence has associated lower vitamin D levels with an elevated risk of developing MS or symptom relapse [95,96,97,98].
Vitamin A			Vitamin A supplement seems to be useful in neuronal plasticity and could improve cognitive ability [105,106].

## Data Availability

Not applicable.

## Abbreviations

The following abbreviations are used in this manuscript:

25(OH)D	25-hydroxyvitamin D
27-OHC	27-hydroxycholesterol
5LO	5-lipoxygenase
AA	Anthranilic acid
AD	Alzheimer’s disease
ADMA	Asymmetric dimethylarginine
ALA	Alpha-lipoic acid
ApoE	Apolipoprotein E
APP	Amyloid precursor protein
Aβ	Amyloid β
BBB	Blood–brain barrier
BHMT	Betaine-homocysteine methyltransferase
CBF	Cerebral blood flow
cECs	Cerebral endothelial cells
CH_2_THF	5,10-Methylenetetrahydrofolate
CH_3_THF	5-methyltetrahydrofolate
CNS	Central nervous system
COMT	Catechol-O-methyltransferase
CSF	Cerebrospinal fluid
DDAH	Dimethylarginine dimethylaminohydodrolase
DMA	Dimethylamine
DMG	Dimethylglycine
DM-PP2Ac	De-methylated PP2A catalytic subunit
DR4/5	Death receptor 4 and 5
EAE	Autoimmune encephalomyelitis
EDSS	Expanded Disability Status Score
ET	Endothelin
FAD	Flavin adenine dinucleotide
HAA	3-hydroxyanthranilic acid
HCs	Healthy controls
HCTL	Homocysteine thiolactone
Hcy	Homocysteine
HHcy	Hyperhomocysteinemia
HK	3-hydroxykynurenine
iNOS	inducible Nitric Oxide Synthase
KA	Kynurenic acid
KAT	Kynurenine aminotransferase
Kyn	kynurenine
KYNU	Kynureninase
MBP	Myelin basic protein
MCI	Mild Cognitive Impairment
MeSe	Methionine synthase
MMA	Methylmalonic acid
MOG	Myelin oligodendrocyte glycoprotein
M-PP2Ac	Methylated PP2A catalytic subunit
MS	Multiple sclerosis
MSSS	Multiple Sclerosis Severity Score
MTHFR	Methylenetetrahydrofolate reductase
NMO	Neuromyelitis optica
NO	Nitric oxide
NOS	Nitric oxide synthase
ONDs	Other neurologic diseases
PD	Parkinson’s disease
PLP	Pyridoxal 5-phosphate
PP2A	Protein phosphatase 2A
PRMT-1	Protein arginine methyltransferases-1
PRMT-4	Protein arginine methyltransferase-4
PS1	Presenilin 1
pS262-tau	Ser262 hyperphosphorylated tau
PTMs	Post-translational modifications
ROS	Reactive oxygen species
RR	Relapsing–remitting
SAH	S-adenosyl homocysteine
SAM	S-adenosyl methionine
SDMA	Symmetric dimethylarginine
SP	Secondary progressive
TCA	Tricarboxylic acid
THF	Tetrahydrofolate
tHcy	Total homocysteine
VCID	Cognitive impairment and dementia
XA	Xanthurenic acid
